# Protein-losing enteropathy in camptodactyly-arthropathy-coxa vara-pericarditis (CACP) syndrome

**DOI:** 10.1186/s12969-016-0093-5

**Published:** 2016-05-25

**Authors:** Bram Peters, Janneke H. M. Schuurs-Hoeijmakers, Joris Fuijkschot, Annette Reimer, Michiel van der Flier, Dorien Lugtenberg, Esther P.A.H. Hoppenreijs

**Affiliations:** Department of Paediatrics, Radboud University Nijmegen Medical Centre, Radboud umc. Geert Grooteplein Zuid 10, Nijmegen, 6525 GA The Netherlands; Department of Human Genetics, Radboud University Nijmegen Medical Centre, Radboud umc. Geert Grooteplein Zuid 10, Nijmegen, 6525 GA The Netherlands; Department Paediatric Cardiology, Radboud University Nijmegen Medical Centre, Radboud umc. Geert Grooteplein Zuid 10, Nijmegen, 6525 GA The Netherlands; Radboud Institute for Molecular Life Sciences, Radboud University Nijmegen Medical Centre, Radboud umc. Geert Grooteplein Zuid 10, Nijmegen, 6525 GA The Netherlands; Paediatric Rheumatology, Radboud University Nijmegen Medical Centre, Radboud umc. Geert Grooteplein Zuid 10, Nijmegen, 6525 GA The Netherlands; Sint Maartenskliniek, Hengstdal 3, Ubbergen, 6574 NA The Netherlands

**Keywords:** Camptodactyly-arthropathy coxa vara-pericarditis (CACP) syndrome, Protein-losing enteropathy (PLE), Hypogammaglobulinaemia, Secondary immunodeficiency, Constrictive pericarditis, Juvenile idiopathic arthritis (JIA), Proteoglycan 4 gene (PRG4), Lubricin, Diagnosis

## Abstract

**Background:**

Camptodactyly-arthropathy-coxa vara-pericarditis (CACP, OMIM: #208250) syndrome is a rare autosomal recessive disease that can be difficult to recognise not only because of its wide clinical variability but also because of its clinical resemblance to juvenile idiopathic arthritis (JIA). PRG4 is the only gene so far known to be associated with CACP syndrome. Children with CACP syndrome lack the glycoprotein lubricin due to recessive mutations in PRG4. Lubricin serves as a lubricant in joints, tendons and visceral cavities (pleural cavity, pericardium) and inhibits synovial proliferation. Children with CACP syndrome suffer from congenital camptodactyly, arthropathy, coxa vara and sometimes pericarditis. This report concerns a child with CACP syndrome complicated by protein-losing enteropathy (PLE), caused by constrictive pericarditis and so contributes to knowledge of the presentation of CACP syndrome.

**Case Presentation:**

A 10- year-old girl with consanguineous parents suffered from congenital camptodactyly and progressive swollen and painful joints. Her father and his sister had similar childhood-onset joint complaints. Laboratory tests showed no signs of inflammation but showed persistent low protein- and IgG- levels, indicating a secondary immunodeficiency. Increased alpha antitrypsin clearance confirmed PLE. Abdominal ultrasound with Doppler showed hepatomegaly and portal hypertension. Echocardiography suggested constrictive pericarditis. However, heart catheterization could not confirm this. Ultrasound and X-ray examination of the joints combined with a puncture of the synovial fluid were performed. These results, combined with the clinical presentation and the consanguinity, suggested CACP syndrome. Due to excessive enteral protein losses, the patient was treated with Cotrimoxazol prophylaxis and immunoglobulin supplements. These supplements were inadequate to achieve normal IgG values. As constrictive pericarditis with subsequent PLE was the best explanation for the excessive IgG losses, pericardiectomy was performed with good results. Genetic testing in our patient was complicated but revealed a pathogenic mutation within the repeat sequence in exon 7 of the PRG4 gene.

**Conclusion:**

PLE resulting from constrictive pericarditis can be a complication of CACP syndrome. As serious complications can arise from the resulting secondary immunodeficiency, we recommend regular evaluation of clinical symptoms of constrictive pericarditis and PLE in children with CACP syndrome.

**Electronic supplementary material:**

The online version of this article (doi:10.1186/s12969-016-0093-5) contains supplementary material, which is available to authorized users.

## Background

CACP syndrome is a rare autosomal recessive disorder characterized by a triad of camptodactyly, childhood non-inflammatory arthropathy with synovial hyperplasia and coxa vara. Occasionally non-inflammatory pericarditis and pleural effusion are present. CACP syndrome has been found in different ethnic populations [1, 2]. Due to its rarity, CACP syndrome is probably under-diagnosed, making it difficult to estimate prevalence rates [1]. Features of CACP syndrome mimic juvenile idiopathic arthritis (JIA), and this may lead to misdiagnosis and incorrect treatment [1, 3].

The pathophysiology of CACP syndrome is not completely known. Patients affected by CACP syndrome lack the glycoprotein lubricin. Its formation involves transcription of the proteoglycan 4 gene (PRG4), which is expressed not only in joints but also in pericardial and pleural cavities as well as the liver, kidneys and skeletal muscles. The PRG4 gene is located on chromosome 1q31.1 (OMIM:*604283). Currently, twenty-two mutations have been reported (HGMD professional 2015.3) [4]. It is predicted that all mutations will lead to a premature stop codon, resulting in the absence of functional lubricin [1–3, 5].

In joints, lubricin is produced by fibroblastic synoviocytes, articular chondrocytes and cells lining articular cartilage and tendon-sheaths, including the covering outer aspects of tendons. Lubricin has lubricating properties and the capacity to regulate cell growth, and it protects the cartilage surface from protein deposits and friction-induced damage [1, 2, 6]. The absence of lubricin causes synovial hyperplasia, which can damage joint cartilage by inhibiting the normal exchange of nutrients and waste products and by invading the articular cartilage surface [7]. Tissue remodelling and calcification of tendon-sheaths may account for the development of camptodactyly [8].

In addition to proliferative synovitis, biopsies of human synovia show deposits of an eosinophilic amorphous material, stromal multinucleated giant cells (originally macrophages) and a paucity of inflammatory cells [6]. The presence of these giant cells is presumably due to the infiltration and accumulation of macrophages, possibly aggravated by the deposit of eosinophilic material, not to proliferation. The exact source of this eosinophilic material is unclear [6]. Synovial fluid in CACP syndrome is viscous, clear, honey-coloured and low in cell count [9].

As fibrosis is observed in pericardial biopsies, it is likely that lubricin serves as an anti-adhesive between the visceral and parietal pericardium. The role of lubricin in the liver, kidneys and skeletal muscles is unclear [2, 10].

The clinical manifestations of CACP syndrome can vary, even within families [3]. The slow progressive onset of CACP syndrome can cause an initially incomplete clinical picture [7]. However, camptodactyly (85–100 %) and arthropathy (100 %) are found consistently [5, 11]. Camptodactyly is symmetrical with a variable distribution. Any finger or toe can be affected. It can be congenital or develop during infancy [3, 11]. Arthropathy is symmetrical and principally involves the large joints (wrists, knees, ankles, elbows and hips). The wrist is the first and most frequently affected joint. The knees are also often affected. Coxa vara is present in 50–90 % of cases, is progressive and becomes more pronounced with age [1, 3, 11]. Spine abnormalities (lordosis, scoliosis and kyphosis) can also occur [3]. Normally, the cervical spine is not affected [9]. Children usually complain of swollen joints, due to hydrops and synovial thickening (without signs of inflammation), joint contractures, restricted movement and sometimes musculoskeletal pains. Non-inflammatory pericarditis occurs in up to 30 % of published cases and has a wide clinical variability, from a self-limiting course to constrictive pericarditis requiring surgical intervention [1, 3, 12].

This report concerns a young female patient with protein-losing enteropathy (PLE) and resultant hypogammaglobulinaemia due to constrictive pericarditis in CACP syndrome. As far as can be established, PLE has never been described as part of the symptom complex of CACP syndrome. Due to this unusual complication, the diagnosis of pericarditis and subsequently CACP syndrome was challenging. This report contributes to knowledge of the presentation of CACP syndrome and provides insight into possible complications.

## Case presentation

**Fig. 1:**
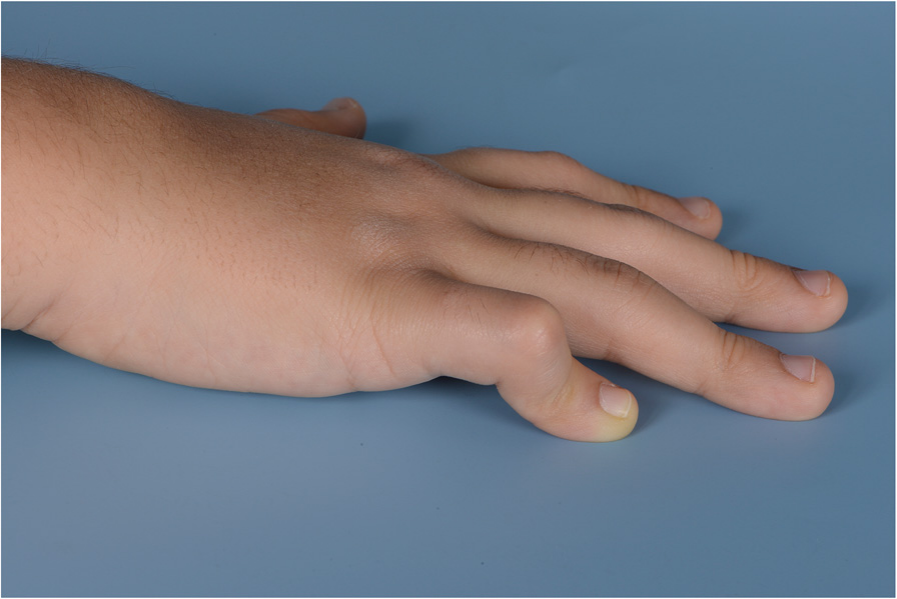
Right hand of the patient. Fifth finger camptodactyly

**Fig. 2:**
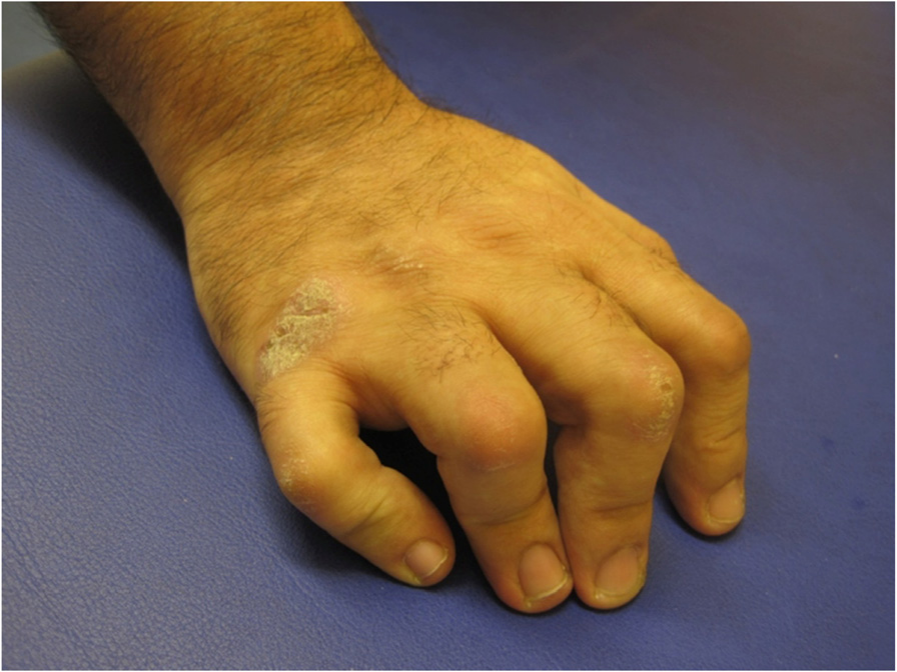
Right hand of the patient's father. Camptodactyly in all fingers. The father has a more extended form of camptodactyly. It demonstrates the variable presentation of the camptodactyly and clinical variability of the CACP syndrome even within families

A ten-year-old girl of Turkish origin with consanguineous parents (for pedigree, see Additional file 1: Figure S1) presented at the paediatric ward of a general hospital with arthralgia. A flexion contracture of the right-hand little finger had been present since early infancy (Fig. 1) and she had been suffering from progressively swollen and painful knees, wrists, elbows and ankles since the age of three. She regularly had a swollen face and in the past she suffered from recurrent respiratory infections. Her father and his sister also experienced similar childhood-onset joint complaints (Fig. 2). The father had been diagnosed with JIA as a child.

On examination, our patient had a camptodactyly of her right fifth finger and a mild curvature of her left index finger. Her metacarpal joints, right wrist, both ankles and both knees were swollen. Extension of both elbows was limited. Iridocyclitis was not present. In view of her joint complaints and recurrent respiratory infections, an autoimmune disease and immunodeficiency were suspected. Laboratory tests showed no signs of inflammation. Reumatoidfactor, ANA and anti-CCP were all negative. Total IgG and total albumin were low (IgG 1.57 g/l and albumin 20 g/l), indicating a secondary immune deficiency.

During a follow-up physical examination, an enlarged liver was found, though no heart murmur was heard. Laboratory tests revealed normal liver enzymes and persistently low IgG with normal IgM and IgA. In CT imaging no intrapulmonary lesions were found. However, pleural and pericardial effusion of unknown origin were present. The girl was referred to our University Medical Centre for further diagnostic tests.

With regard to protein loss, faecal analysis showed an increased alpha antitrypsin clearance, suggesting PLE. An abdominal ultrasound with Doppler was conducted and showed hepatomegaly and portal hypertension (reversed flow in portal vein). A full cardiac analysis was consequently carried out. The electrocardiogram was normal. Echocardiography showed moderate pericardial effusion and a septal diastolic bounce, suggesting constrictive pericarditis. The Doppler measurements were not conclusive. Because we could not confirm a cardiac origin of the portal hypertension, we decided to exclude a hepatogenic origin of the portal hypertension first. A percutaneous liver biopsy revealed a-specific fibrosis interpreted as chronic venous congestion. To determine intra-cardiac pressure to confirm constrictive pericarditis, heart catheterization was then performed, which showed elevated venous pressures (mean 22 mm of mercury), equalization of end-diastolic pressures in all cardiac chambers and a right ventricular pressure of 31 mm of mercury (upper limit of normal). Kussmaul’s sign was positive. As the presence of the square root sign of the right ventricle was inconsistent and enhanced ventricular interdependence was absent, we could not confirm the diagnosis of constrictive pericarditis. Moreover, a thickened pericardium was not seen on a subsequent cardiac MRI.

Since the diagnosis of constrictive pericarditis could not be confirmed, other diagnoses were reconsidered. Investigations to rule out JIA and other autoimmune diseases causing polyarthritis were undertaken, including an ultrasound of the joints and a puncture of the synovial fluid. The synovial capsule seemed thickened. The synovial fluid was mildly honey-coloured and showed some multinucleated macrophages (CD68 positive) without signs of inflammation. Inflammatory arthritis was thus excluded. CACP syndrome was suggested and further evaluation was performed. X-ray examination showed coxa vara, peri-articular osteopenia and flattened metacarpal and phalangeal joints. As the overall clinical presentation and results were suggestive of CACP syndrome, the girl and her father were referred for genetic testing and counselling. Meanwhile the patient was treated with immunoglobulin supplements and Cotrimoxazol prophylaxis. Because high doses of IgG were needed to compensate for her losses (1.6 g/kg/28 days), intravenous administration was chosen for better tolerance of treatment despite potential pharmacokinetic disadvantages. However, due to excessive enteral protein loss, normal serum IgG values could not be achieved. As constrictive pericarditis with subsequent PLE was the best explanation for the excessive IgG losses, this indicated a pericardiectomy. During this procedure a thickened parietal epicardium and fibrotic visceral epicardial layer on the right and left ventricle were removed, after which central venous pressure dropped from 14 to 7 mm of mercury. After this intervention the PLE and portal hypertension were resolved quickly. Supplementation of intravenous immunoglobulins was discontinued successfully. Repetitive measurements of serum albumin and IgG were all within normal ranges, indicating a full stop of the PLE. One year after the pericardiectomy, all echocardiographic measurements were normal, meaning no effusion, normal wall motion and normal Doppler measurements.

Clinically, the girl is in good condition. Besides the camptodactyly and limited extension of her elbow, no other joint complains were left. Additionally, the oedema and joint swellings disappeared.

With regard to the genetic testing, the genome-wide array analysis (Affymetrix CytoScan HD array platform) of the girl and her father showed a total of 83 Mb of shared homozygosity, including the PRG4 gene (see Additional file 2: Figure S2). The homozygous regions did not contain other candidate genes likely to explain the phenotype. Sanger sequence analysis of the coding sequence of PRG4 was performed on the DNA of the girl in our laboratory (NM_005807.3). All exons in the gene were eventually tested. Sixty percent of the PRG4 gene was sequenced and analysed successfully by conventional PCR and Sanger sequencing, including complete exons 1–6 and 8–13. As the analysis of exon 7 encountered technical problems and no pathogenic mutation was found in the PRG4 gene, diagnostic exome sequencing was carried out essentially as previously described in literature (Genome Diagnostics Radboudumc, gene package ‘multiple congenital anomalies’, version DG2.3×) [13]. A homozygous pathogenic one basepair deletion, c.1290del (p. (Thr431fs), was identified in exon 7 of the PRG4 gene, resulting in a premature stop codon (see Additional file 3: Figure S3). No other pathogenic mutations were identified in the disease gene package.

## Discussion

This report concerns a girl with CACP syndrome complicated by PLE as a consequence of constrictive pericarditis. The diagnostic process was challenging for a number of reasons.

PLE has several causes. With constrictive pericarditis, elevated central venous pressure causes congestion of the intestinal lymphatic drainage, leading to the direct leakage of proteins, including IgG, into the intestinal lumen [14]. Constrictive pericarditis usually results in hemodynamic changes that are related to fluid overload (e.g. peripheral oedema, elevated central venous pressure, hepatomegaly and pleural effusion), decreased cardiac output (e.g. fatigue, dyspnea on exertion) and an abnormal thickening of the pericardium >3 mm [14, 15]. However, in this case, PLE was the only principal sign of constrictive pericarditis. Hypoproteinemia was the most likely cause of the pleural effusion and peripheral oedema.

Additionally, our patient had joint complaints that were similar to JIA. As no signs of inflammation were found in the blood or the synovial fluid, JIA was unlikely to be the cause of these joint complaints. Family history, consanguinity and early-onset camptodactyly provided significant clues for the diagnosis of this case [11, 12]. Radiological examinations also assisted greatly. Several distinguishing features characterise CACP syndrome: periarticular osteopenia with increased joint space in the affected joints, squaring or flattening of the metacarpal and phalangeal heads, coxa vara with short femoral necks and flat, irregular femoral heads and, occasionally, highly distinguishing intraosseus fluid-filled herniations of the acetabulum [1, 9, 11, 12]. Radiologic features of CACP can mimic the acute non-erosive phase of JIA [9, 12]. However, in a more developed stage, inflammation caused by JIA results in the destruction of the joint and the narrowing of the joint space. These types of erosion are not seen in CACP syndrome [9].

New insights into the role of lubricin in arthralgia reveal that CACP syndrome is more related to rheumatoid arthritis (RA) and osteoarthritis (OA) than previously thought. In humans with RA or OA and in animal joint injuries, changes in lubricin’s abundance and/or function have been found. This acquired form of lubricin deficiency in RA and OA provides new insights into the protective function of lubricin in arthralgia and explains how CACP syndrome mimics clinical features of JIA [8, 10, 16].

The girl was given a clinical diagnosis of CACP syndrome. Subsequent extensive genetic testing was performed to confirm the clinical diagnosis. Exome sequencing identified a homozygous pathogenic mutation, which was identified in exon 7 of the PRG4 gene. This mutation is also predicted to lead to a premature stop codon, resulting in the absence of functional lubricin. Because the mutation was located in the part of exon 7 that failed amplification, it had not been previously detected by Sanger sequencing. This part of exon 7 (c.1128-2733) was unsuccessfully amplified by Sanger sequencing due to its repetitive nature, as mentioned elsewhere [7]. To the best of our knowledge, this mutation has not been reported before. Confirming the carrier status of the mother would have further supported the hereditary nature of the mutation that was found. Since in our laboratory exome sequencing is the only technique to investigate exon 7, we have not been able to confirm the affected status of the father and the carrier status of the mother through an affordable technique. Although there is no direct proof that the mutation in the girl originates from both parents, the consanguinity (see the pedigree in Additional file 1: Figure S1) and the shared homozygous region of the girl and her father are indirect evidence that the girl inherited this mutation from her parents.

From a therapeutic perspective, there are no accounts yet of effective arthropathy treatment for children with CACP syndrome. In literature two cases are reported in which a total hip arthroplasty was carried out in response to severe disability problems. This intervention resulted in the relief of pain and improvement of function [17]. CACP symptoms do not respond to anti-inflammatory drugs. In general, patients may benefit from vitamin D and calcium supplementation [1]. There are indications that the intra-articular injection of recombinant lubricin can prevent synovial thickening and the degeneration of cartilage in OA and CACP syndrome. Rhee et al. have demonstrated that purified and recombinant lubricin in vitro prevents protein deposition onto cartilage surface and inhibits the adhesion-dependent cell growth of synoviocytes in lubricin-mutant mice [8]. Flannery et al. evaluated the intra-articular administration of a purified preparation of recombinant lubricin versus a phosphate-buffered saline in an experimental rat model of OA. In this model, the medial meniscus was cut through the full thickness, causing a complete rupture. After 4 weeks of treatment, local administration of lubricin had a significant disease-modifying and chondro-protective effect on the progression of OA [18]. Recombinant lubricin may feature in future treatment options for CACP patients, although clinical studies are required and technical problems regarding local delivery must be overcome [16].

## Conclusion

To the best of our knowledge, this is the first account of a case of massive PLE resulting from constrictive pericarditis that presents as a complication in a patient with CACP syndrome. Exome sequencing revealed a pathogenic mutation within the repeat sequence in exon 7 of the PRG4 gene, a region that failed amplification by PCR and Sanger sequencing. It is possible for PLE to be the only sign of constrictive pericarditis. As serious complications can arise from the resulting secondary immunodeficiency, it is advisable to examine children with CACP syndrome regularly for clinical signs of PLE and constrictive pericarditis. In the case of a positive result, a referral for full cardiac examination and an evaluation of immunoglobulins is recommended.

## Consent

Written informed consent was obtained from the parents of the patient for the publication of this case report, including associated images. A copy of the written consent is available for review by the Editor-in-Chief of this journal.

## Additional files

Additional file 1: Figure S1. Pedigree showing consanguinity. Index is indicated with the arrow. Individuals in black suffer from arthropathy (starting early in childhood) and camptodactyly. (DOCX 68 kb) Additional file 2: Figure S2. Shared regions of homozygosity. Table shows the shared homozygous regions between the patient and her father and the number of OMIM autosomal recessive disease genes that reside within these homozygous regions. The two largest regions of homozygosity are a 45 Mb homozygous region on chromosome 1q22q32.1—including PRG4—and a 24 Mb homozygous region on chromosome 8. (DOCX 63 kb) Additional file 3: Figure S3. Graphical overview of the genomic structure of PRG4 (NM_005807.4). (A) Red stripes indicate mutations that are described in the Human Gene Mutation Database. Sequence depth is visualized in the lower part in light gray. Note the difficult-to-sequence repeat region of exon 7, indicated by less sequence depth. (B) Zoom-in of the BAM-files of our patient, showing the reads containing the homozygous mutation c.1290del (arrow). Diagnostic exome sequencing was performed, as described in detail by Neveling et al., using an Illumina HiSeq2000TM sequencer at BGI-Europe (Copenhagen, Denmark) [13]. Read alignment to the human reference genome (GrCH37/hg19) and variant calling was performed at BGI using BWA and GATK software, respectively. Variant annotation was performed using a custom-designed in-house annotation and variant prioritization pipeline. All PRG4 exons were analyzed using this test. (PPTX 731 kb)

## Abbreviations

ANA: anti-nuclear antibodies
Anti-CCP: anti cyclic citrullinated peptides
CACP syndrome: camptodactyly-arthropathy-coxa vara-pericarditis syndrome
CT: computer tomography
IgG, IgM, IgA: immunoglobulin G, M, A
JIA: juvenile idiopathic arthritis
MRI: magnetic resonance imaging
OA: osteoarthritis
OMIM: online mendelian inheritance in man
PCR: polymerase chain reaction
PLE: protein losing enteropathy
PRG4 gene: proteoglycan 4 gene
RA: rheumatoid arthritis
