# Supplementary figures and images for: Protein-losing enteropathy in camptodactyly-arthropathy-coxa vara-pericarditis (CACP) syndrome
Source: Pediatr Rheumatol Online J. 2016 May 25;14:32. doi: 10.1186/s12969-016-0093-5 (PMC4880819; doi:10.1186/s12969-016-0093-5)

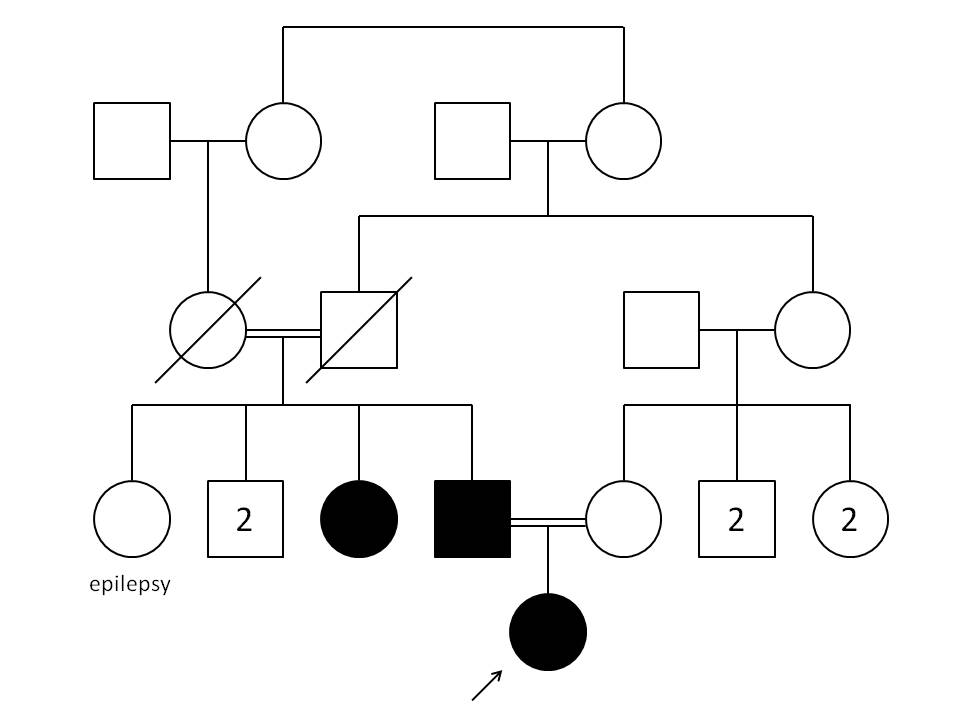

Supplement: Additional file 1: Figure S1. — Pedigree showing consanguinity. Index is indicated with the arrow. Individuals in black suffer from arthropathy (starting early in childhood) and camptodactyly. (DOCX 68 kb) [file 12969_2016_93_MOESM1_ESM.docx]

## Slide 1
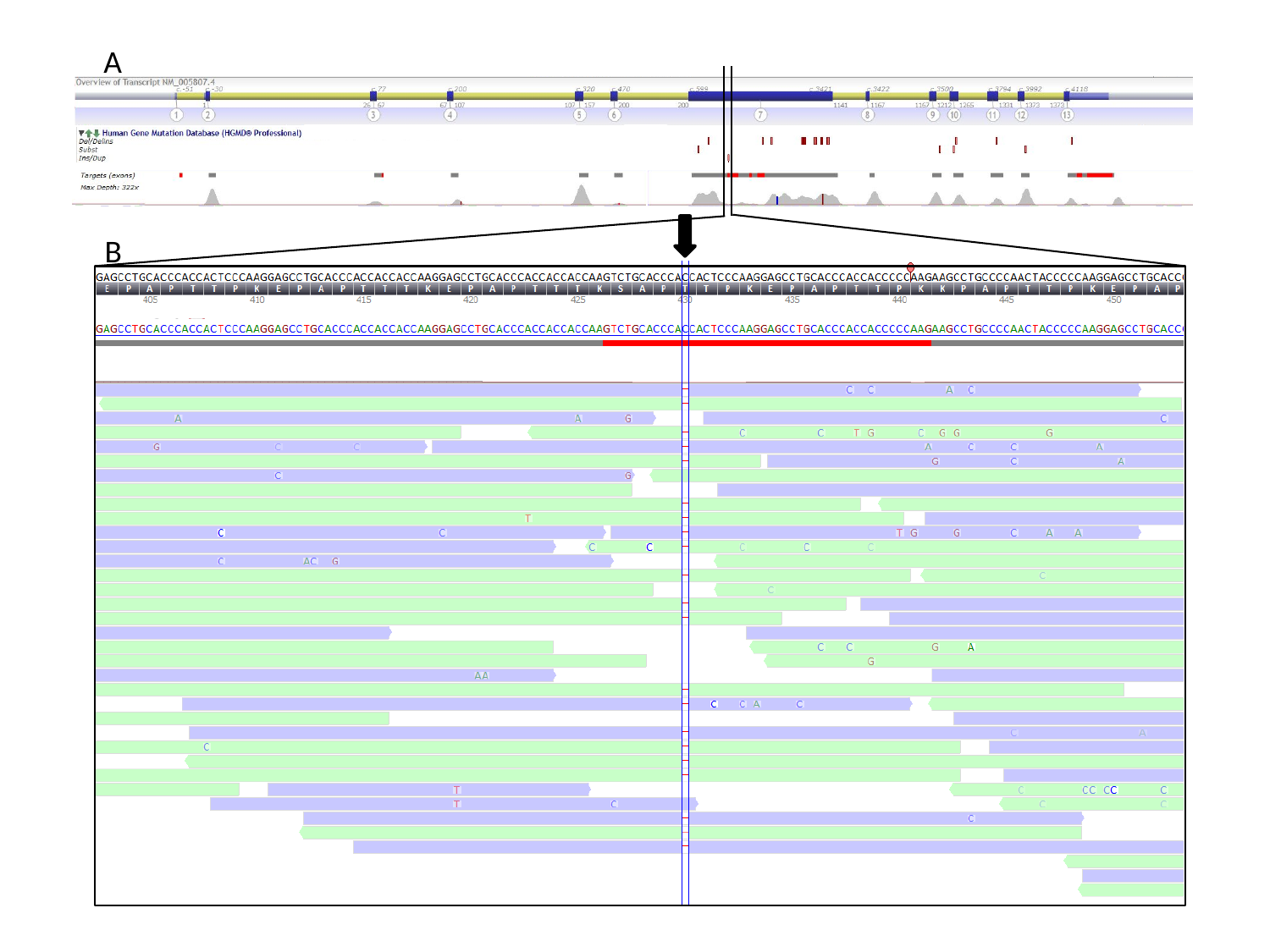

A
B

Supplement: Additional file 3: Figure S3. — Graphical overview of the genomic structure of PRG4 (NM_005807.4). (A) Red stripes indicate mutations that are described in the Human Gene Mutation Database. Sequence depth is visualized in the lower part in light gray. Note the difficult-to-sequence repeat region of exon 7, indicated by less sequence depth. (B) Zoom-in of the BAM-files of our patient, showing the reads containing the homozygous mutation c.1290del (arrow). Diagnostic exome sequencing was performed, as described in detail by Neveling et al., using an Illumina HiSeq2000TM sequencer at BGI-Europe (Copenhagen, Denmark) [13]. Read alignment to the human reference genome (GrCH37/hg19) and variant calling was performed at BGI using BWA and GATK software, respectively. Variant annotation was performed using a custom-designed in-house annotation and variant prioritization pipeline. All PRG4 exons were analyzed using this test. (PPTX 731 kb) [file 12969_2016_93_MOESM3_ESM.pptx]
