# Supplementary material for: Protein-losing enteropathy in camptodactyly-arthropathy-coxa vara-pericarditis (CACP) syndrome
Source: Pediatr Rheumatol Online J. 2016 May 25;14:32. doi: 10.1186/s12969-016-0093-5 (PMC4880819; doi:10.1186/s12969-016-0093-5)
Supplement: Additional file 2: Figure S2. — Shared regions of homozygosity. Table shows the shared homozygous regions between the patient and her father and the number of OMIM autosomal recessive disease genes that reside within these homozygous regions. The two largest regions of homozygosity are a 45 Mb homozygous region on chromosome 1q22q32.1—including PRG4—and a 24 Mb homozygous region on chromosome 8. (DOCX 63 kb) [file 12969_2016_93_MOESM2_ESM.docx]

|  | **Shared regions of homozygosity** | **OMIM AR Genes** | **Length (Mb)** |
| --- | --- | --- | --- |
| 1 | chr1:155971759-200984022 | 36 | 45,01 |
| 2 | chr3:62613-2755427 | 0 | 2,69 |
| 3 | chr6:103484830-107186184 | 0 | 3,70 |
| 4 | chr8:72695848-96596132 | 11 | 23,90 |
| 5 | chr11:37234831-39282920 | 0 | 2,058 |
| 6 | chr12:125486796-130660879 | 0 | 5,17 |
| Total |  |  | 82,53 |
